# Treatment of triple-negative breast cancer with Chinese herbal medicine: A prospective cohort study protocol

**DOI:** 10.1097/MD.0000000000008408

**Published:** 2017-11-03

**Authors:** Hui Meng, Nan Peng, Mingwei Yu, Xu Sun, Yunfei Ma, Guowang Yang, Xiaomin Wang

**Affiliations:** a School of Graduates, Beijing University of Chinese Medicine; b Oncology Department, Beijing Chinese Medicine Hospital Affiliated to Capital Medical University, Beijing, China.

**Keywords:** Chinese herbal medicine, quality of life, study design, disease-free survival, triple-negative breast cancer

## Abstract

**Introduction:**

Triple-negative breast cancer (TNBC) is featured with the biological properties of strong aggressive behaviors, rapid disease progression, high risk of recurrence and metastasis, and low disease free survival. Patients with this tumor are insensitive to the endocrine therapy and target treatment for HER-2; therefore, chemotherapy is often used as routine treatment in clinical. Because of the fact that a considerable number of patients seek for Chinese herbal medicine (CHM) treatment after operation and chemotherapy and (or) radiotherapy, it is thus need to evaluate the correlation between Chinese herbal medicine treatment and prognosis.

**Methods and analysis:**

This is a multicenter, prospective cohort study started in March 2016 in Beijing. A simple of 220 participants diagnosed with TNBC were recruited from nine hospitals and are followed up every 3 to 6 months till March 2020. Detailed information of participants includes personal information, history of cancer, quality of life, symptoms of traditional Chinese medicine and fatigue status is taken face-to-face at baseline.

**Ethics and dissemination:**

The study has received ethical approval from the Research Ethical Committee of Beijing Hospital of Traditional Chinese Medicine affiliated to Capital Medical University (No.2016BL-014-01). Articles summarizing the primary results and ancillary analyses will be published in peer-reviewed journals.

**Trial registration:**

Chinese Clinical Trial Registry: ChiCTR-OOC-16008246.

## 1 Introduction

Triple-negative breast cancer(TNBC) is defined by an immunohistochemical absence of expression for estrogen receptor (ER), progesterone receptor (PR), and human epidermal growth factor receptor-2 (HER-2),^[1]^ it accounts for about 10% to 15% of breast carcinomas.^[2]^ The disease shows the characteristic of highly invasive and malignant, prone to an early pattern of recurrence and metastasis.^[3]^ TNBC is a collection of different breast cancer that is still poorly characterized at molecular level, and lack of definitive prognostic markers and selective targets of therapy.^[4,5]^ At present, chemotherapy remains the predominant systemic treatment for TNBC patients in both the early and advanced-stages of the disease and data from many studies showing significant benefit of chemotherapy.^[6,7]^ There is no proven targeted therapies for TNBC and the patients are of poor prognosis. Based on the above reasons, TNBC becomes a hot spot in the research of breast cancer.

Traditional Chinese medicine (TCM) has a history of thousands of years and can adjust the body in balance to prevent the recurrence/metastasis of cancers.^[8]^ CHM based on TCM has been increasingly used over the past decades around the world and has become well known for its significant role in enhancing efficacy and reducing toxicity during chemotherapy,^[9]^ and also has a certain effect in preventing and treating cancer.^[10]^ It is estimated that the United States National Cancer Institute (NCI) spends around $120 million each year on TCM related research projects.^[11]^ TNBC patients and their caregivers often consider CHM as a therapeutic method besides operation and chemotherapy/radiotherapy. Relevant article^[12]^ reports that CHM plays an important role in the combined, consolidate and maintenance treatment of TNBC.

## 2 Objectives

### 2.1 Primary

The primary objective of this study is to explore the association of CHM and recurrence and metastasis rate.

### 2.2 Secondary

1. To investigate the relationship between the Quality of life (QoL) and the use of CHM in patients with TNBC. We evaluated the QoL in TNCB patients with Functional Assessment of Cancer Therapy (FACT) Scale and Eastern Cooperative Oncology Group (ECOG) criterion.
2. To evaluation the correlation between breast cancer related symptoms and the use of CHM. Breast cancer related symptoms include fatigue, sleep quality, depression, and anxiety.

## 3 Methods and analysis

### 3.1 Study design

This study is a multicenter, prospective cohort study. The study has started in March 2016 and is currently ongoing. Two hundred and twenty TNBC patients aged between 18 to 75 years with II-III stage after surgery are enrolled from 9 hospitals. All participants need to complete the standard chemotherapy and (or) radiotherapy specified by the National Comprehensive Cancer Network (NCCN) guidelines according to their disease states. At the time of the participants entered the study, the duration of the last chemotherapy and (or) radiotherapy was not more than half a year. The whole study consists of 2 stages, stage I comprises a cross-sectional study-baseline and stage II is a cohort follow-up study across a 3-year period. Participants will be followed up every 3 to 6 months till recurrence or metastasis for 3 years. The feasibility and preciseness of the whole process were supervised by 1 to 2 supervisors.

### 3.2 Study settings and participants

**Figure 1:**
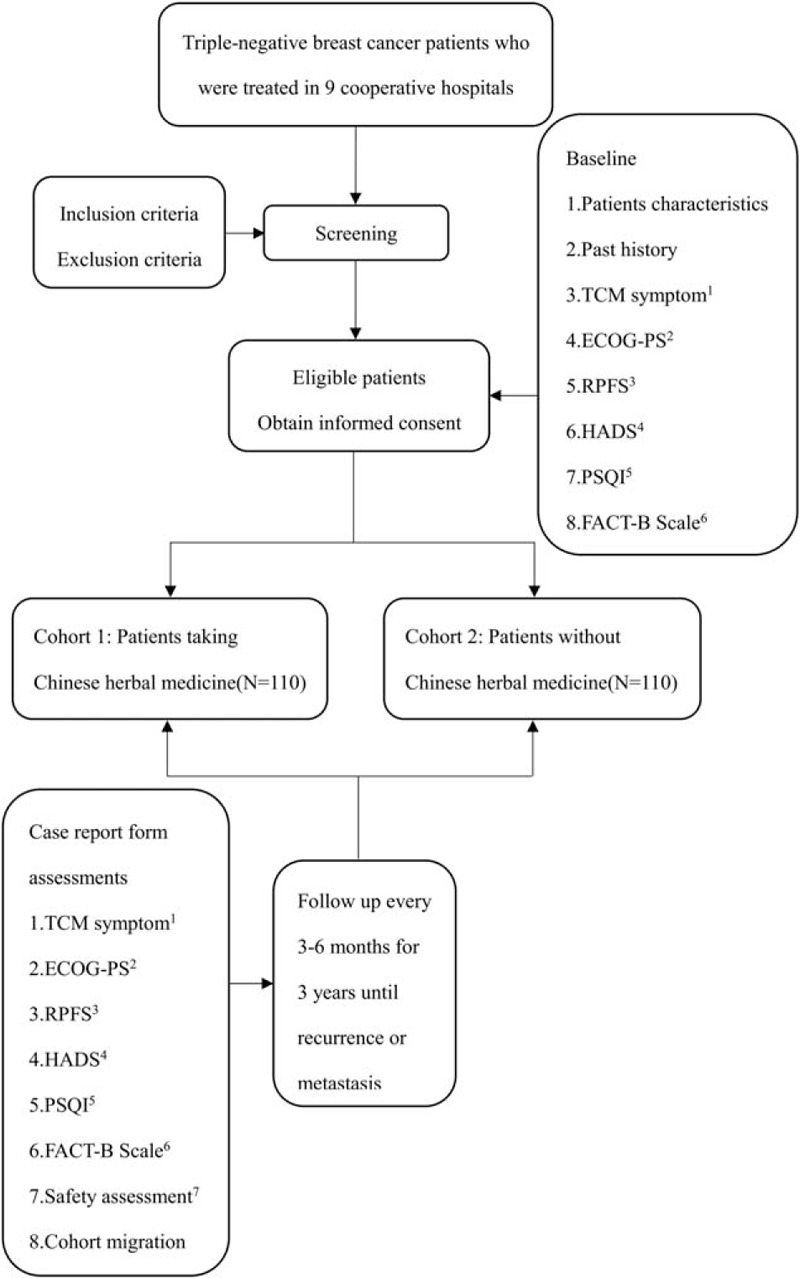
Project overview. TCM symptom^1^: qi deficiency, palpitation, dysphoria in chest and palms. ECOG-PS^2^: Eastern Cooperative Oncology Group Performance Status. RPFS^3^: Revised Piper Fatigue Scale. HADS^4^: Hospital Anxiety and Depression Scale. PSQI^5^: Pittsburgh sleep Quality Index. FACT-B Scale^6^: Functional Assessment of Cancer Therapy-B Scale. Safety assessment^7^: Computed Tomography/Brightness mode/Magnetic Resonance Imaging/Bone Scan, tumor markers (CEA, CA125, CA153), blood routine test, routine urine test, stool routine test, liver/kidney function, electrocardiogram. CRF = Case Report Form, TCM = traditional Chinese medicine.

The study is being performed in 9 hospitals in Beijing include Beijing traditional Chinese Medicine Hospital affiliate to Capital Medical University, China-Japan Friendship Hospital, People's Hospital of Peking University, Chinese PLA General Hospital, Cancer Hospital of Chinese Academy of Medical Science, Guang’Anmen Hospital of the China Academy of Traditional Chinese Medicine, Beijing Tiantan Hospital affiliate to Capital Medical University, Beijing Luhe Hospital affiliate to Capital Medical University, and Beijing Shijitan Hospital affiliate to Capital Medical University. All TNBC patients who came to the above hospitals should be screened. For those eligible patients, researchers will give her and her guardian the detailed description of this study. After obtained the informed consent of the patient and her family, she was enrolled in the study. Participants taking CHM in 1 of the above 9 hospitals were referred to as cohort 1 and patients received chemotherapy or radiotherapy without CHM belong to cohort 2. Doctors of traditional Chinese medicine in the above hospitals prescribe CHM for patients according to their clinical symptoms, tongue and pulse, doctors at the above nine hospitals will also provide life guidance for these TNBC patients. The study schema and estimated recruitment numbers are presented in Figure 1. Inclusion and Exclusion criteria are presented in Table 1. Two investigators will be responsible for the follow-up of each participant at baseline and every 3 to 6months, data collection schedule is presented in Table 2.

**Table 1:**
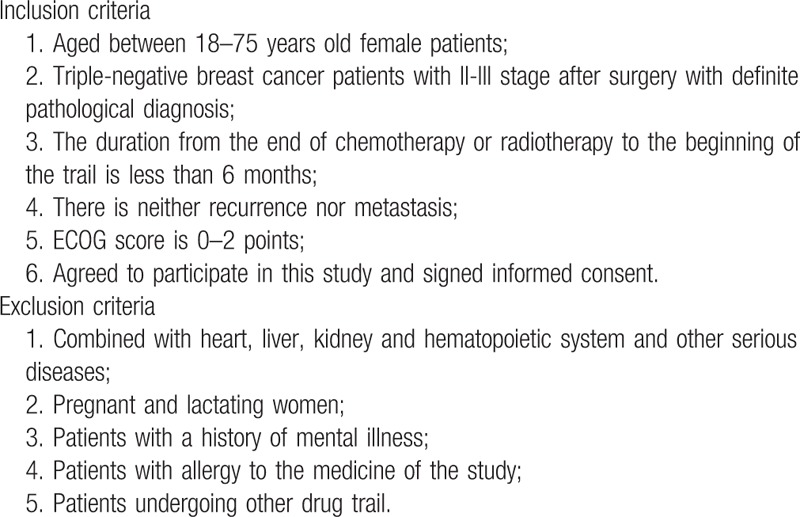
Inclusion/exclusion criteria.

**Table 2:**
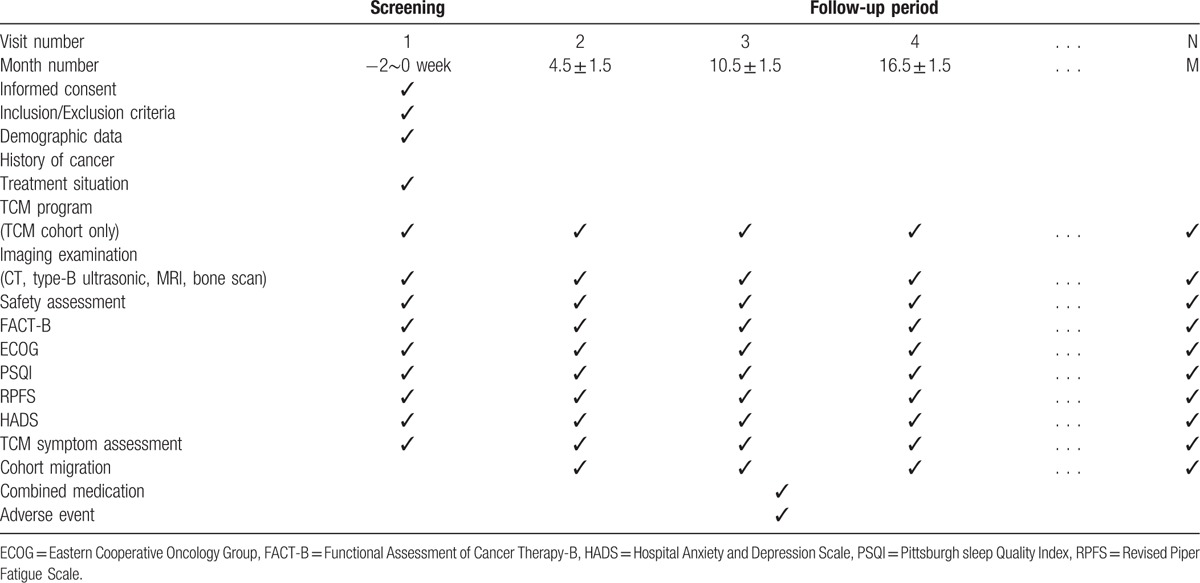
Data collection schedule.

### 3.3 Ethical issues

The study registered in the Chinese clinical trial registry (No. ChiCTR-OOC-16008246) and received ethical approval from the Research Ethical Committee of Beijing Hospital of Traditional Chinese Medicine Affiliated to Capital Medical University (No.2016BL-014-01). Eligible patients participate in the study after obtained an informed consent.

### 3.4 Outcomes

The primary outcomes measure are as follows:

#### 3.4.1 Primary outcomes

The primary outcome of this study is 3-year disease-free survival and 3-year disease-free survival rate.

#### 3.4.2 Secondary outcomes

1. To elucidate the correlation between the QoL and the use of CHM in patients with TNBC. We evaluated the QoL in TNCB patients with FACT Scale and ECOG criterion. FACT scale is a common form of a cancer treatment function evaluation system of the QoL in cancer patients developed by Cella et al.^[13]^ It is constituted by general version (FACT-G) for measuring the quality of life of cancer patients and specific scale in specific cancer. FACT-G consists of 27 entries and is divided into 4 quarters: these are physical well-being, social/family well-being, emotional well-being, and functional well-being. FACT-B adds the specific modules of breast cancer based on FACT-G. Each entry is set in a hierarchical setting (0–4), the higher the score, the better the quality of life. The ECOG criterion is developed by ECOG.^[13]^ The standardized criteria are highly detailed and used to evaluate the toxicity criteria, response criteria and definitions of response by organ site involvement. It is set to 0 to 5 score, the higher the score, the worse the physical condition.
2. To evaluate the correlation between fatigue, sleep quality, depression, anxiety, and the use of CHM. Most TNBC patients exhibit symptoms associated with breast cancer such as fatigue, sleep disorders, depression, and anxiety. We selected Piper Fatigue Scale (PFS), Pittsburgh Sleep Quality Index (PSQI), and Hospital Anxiety and Depression Scale (HADS) to assess the extent of these symptoms. PSQI is a self-related questionnaire to evaluate the sleep quality in the past few months and is easy to understand and self rating.^[14]^ It consists of 19 self-assessments and 5 peer-assessments entries, scoring only 19 self-assessment questions. The total score ranges from 0 to 21, the higher the score, the worse the sleep quality. Fatigue is one of the frequently occurring symptoms in cancer patients,^[15]^ TNBC patients are no exception. Wu HS et al^[16]^ first use PFS in evaluating cancer-related fatigue(CRF). PFS includes 22 entries, respectively measuring from 4 aspects, such as behavior, seriousness, emotion, feeling and cognition, and sentiment. The 22 items are digital representation of 0 to 10, 0 indicates no and 10 indicates very serious. The higher the score, the heavier the tiredness. HADS was devised by Zigmond and Snaitha about 30 years ago,^[17]^ it is a simple and convenient clinical tool to analyze the subjective symptoms of patients. It consists of 14 items and each item sets 0 to 4 score, the higher the score, the severer the depression.

Each participant has a case report form and all relevant data will be recorded in it, 2 clinical research associates supervise the whole research process.

### 3.5 Sample size calculation

The primary objective of the research is 3-year DFS rate and we use it as the basis for the calculation of sample size. Referring to the relevant large-scale cohort study,^[18]^ the 3-year DFS rate of TNBC patients after surgery is about 77%, we assume it to be increased to 86%^[19]^ with the treatment of CHM. The sample size calculation formula for cohort study is n = 2p(1-p)(uα + uβ)/(p1-p2),^[2]^ p = (p1 + p2)/2, α assign to 0.05 and β assign to 0.2, the power is 80%, we need 92 participants of each cohort. The drop-out rate is considered as 20%, then the sample size of each cohort is 110 and the total sample size is 220.

### 3.6 Safety assessments

Although focusing on prognosis and the QoL of TNBC patients with CHM, the safety of taking CHM is also the focus of attention. Blood routine test, urine routine test, stool routine test, liver and kidney function test were registered at baseline, and each follow-up visit to evaluate whether there is any functional damage after taking CHM. Morevover, tumor makers (CEA, CA125, CA153) were checked in to estimate whether CHM promotes tumor progression. In addition, investigators will ask participants at the time of each follow-up if they have any adverse effects (AEs) include physical discomfort or impairment throughout the course study. If there were any AEs, the investigator would provide appropriate treatment to the patient immediately and record it in the case report form (CRF^∗∗^) in time. At the same time, the severity of the AE and the relationship with CHM should be evaluated. If the AE is related to CHM or had an impact on the patient, the CHM should be stopped immediately and that patient should be closely observed. Meanwhile, details should be reported to the supervisor and recorded in the patient's file.

### 3.7 Observation time

Primary endpoint: Local recurrence or distant metastasis.

Study endpoint: Local recurrence or distant metastasis, death, or the end of the study.

### 3.8 Statistical analysis

All statistical analysis will be performed using Statistical Packages of Social Sciences software (SPSS) 19.0. Statistical significance will be defined as the *P* < 0.05. Based on the data distribution, the measurement data will be analyzed by ANOVA or *U* Mann-Whitney test, counting data using *χ*^*2*^test or *U* Mann-Whitney test. The survival data will be analyzed by Kaplan-Meier method in 1, 2, 3-year DFS rate and the recurrence and metastasis rate, then draw the survival curve. The comparison of survival curves between the two cohorts is based on Log-rank method. Repeated ANOVA will be used to evaluate the repeated measures such as the QoL, physical condition, and symptoms related to breast cancer. Evaluation of therapeutic efficiency will be analyzed by the data of full analysis set (FAS) and per protocol set (PPS), safety evaluation will use the data of safety analysis set (SS) for statistical analysis.

## 4 Discussion

CHM has been used for thousands of years in China. In recent years, it has been gradually accepted by Western countries.^[20]^ Thus far, literatures^[21,22]^ reported that CHM is widely used to reduce the side effects of chemotherapy and increase survival of patients, antitumor immune response and the general state of the patients. Also, research^[23]^ reported that CHM combined with chemotherapy can improve survival rate and the quality of life. To evaluate the relationship between CHM and tumor recurrence and metastasis, we choose a simple disease TNBC as the research type. So far it has no other therapeutic regimen besides chemotherapy and radiotherapy after surgery and we can eliminate interference from other treatment factors.

There are several strengths in our study. First, this is the first multicenter prospective observational cohort study on CHM in TNBC patients. There are 9 hospitals participating in this study. It can provide the evidence both relatively objective and consistent with the clinical practice of CHM intervention for TNBC. Second, we choose the observational study design as it is more humanistic than randomized controlled trial (RCT). Although RCT is recognized as the highest level of evidence in the field of evidence based medicine (EBM), it faces a series of ethical challenges for patients with cancer, especially for the research related to survival time. However, the observational cohort study greatly values individual willingness for the choices of treatment and maximize the applicability of CHM in clinical practice.^[24]^ Third, doctors write out a prescription based on the patient's TCM syndrome differentiation^[25]^ instead of roughly dividing into a few types of syndrome. This improves not only the accuracy of syndrome differentiation, but also the pertinence of CHM. Lastly, the study will produce a scroll queue of TNBC that is not only used for this study, but the relevant information from the rolling queue will continue to be followed up and recorded to provide effective and detailed information for the subsequent researches.

Some limitations of this study are as follows. In the field EBM, the cohort study is an observational study and the evidence level is lower than RCT.^[26]^ But after weighing the research program and the patients’ interests, we choose to maximize the latter one to use a cohort study. In addition, there may be an imbalance baseline condition between the two cohorts. To resolve the problem, we will analyze the data at different levels according to different stages of disease, age, and other possible related factors. Since it is a cohort study, queue migration is inevitable. Strengthening the management of follow-up of participants is one of the effective ways to avoid the problem. Patients will be followed up every 3 to 6 months, their medication and physical conditions will be recorded in detail in the CRF. Finally, there are only 220 TNBC patients will be recruit and the sample size looks small for a prospective cohort study. Yet it is limited by research period and funding. Nevertheless, the observation for the TNBC participants take part in this study won’t be stopped three years later. We will continue to follow up these participants to get a long-term information and then to analyze and evaluate the effect of TCM on TNBC more accurately.
